# Single-Water Microsolvation Promotes Proton Sharing in a Neutral Formic Acid–Ammonia Complex

**DOI:** 10.1021/jacs.6c13418

**Published:** 2026-10-12

**Authors:** Fan Xie, Luigi Crisci, Wenhao Sun, Federico Lazzari, Vincenzo Barone, Melanie Schnell

**Affiliations:** † Hefei National Research Center for Physical Sciences at the Microscale, 12652 University of Science and Technology of China, Hefei, Anhui 230026, China; ‡ 28332 Deutsches Elektronen-Synchrotron DESY, Notkestr. 85, 22607 Hamburg, Germany; § 19004 Scuola Superiore Meridionale, Largo San Marcellino 10, Napoli 80138, Italy; ∥ 563196 INSTM, via G. Giusti 9, Firenze 50121, Italy; ⊥ Institut für Physikalische Chemie, Christian-Albrechts-Universität zu Kiel, Max-Eyth-Str. 1, 24118 Kiel , Germany

## Abstract

Can single-water microsolvation promote proton sharing in a neutral acid–base complex? We address this question for formic acid–ammonia by isolating its monohydrated complex with broadband rotational spectroscopy and interpreting the resolved ^14^N hyperfine structure with calibrated high-level quantum chemistry. The observed complex is a single dominant HCOOH–NH_3_–H_2_O isomer with effective *C*_*s*_ symmetry, in which NH_3_ remains the primary proton acceptor, while water closes a cooperative hydrogen-bond cycle by accepting from NH_3_ and donating to the carbonyl oxygen. Rather than simply stabilizing the binary acid–base contact, this water molecule measurably increases its proton-sharing character. A resolved A/E tunneling pattern from NH_3_ internal rotation supplies the missing orientation needed to rotate the experimental ^14^N quadrupole tensor into the local ammonia frame, yielding an experimental χ_*zz*_ component and a quadrupole-derived Townes–Dailey ionicity of approximately 26%. High-level structures show the same directional response: O–H elongation, H···N contraction, and O···N contraction relative to HCOOH–NH_3_. Together, these results show that one water molecule can promote proton sharing in a neutral formic acid–ammonia complex without producing a separated ion pair.

## Graphical abstract

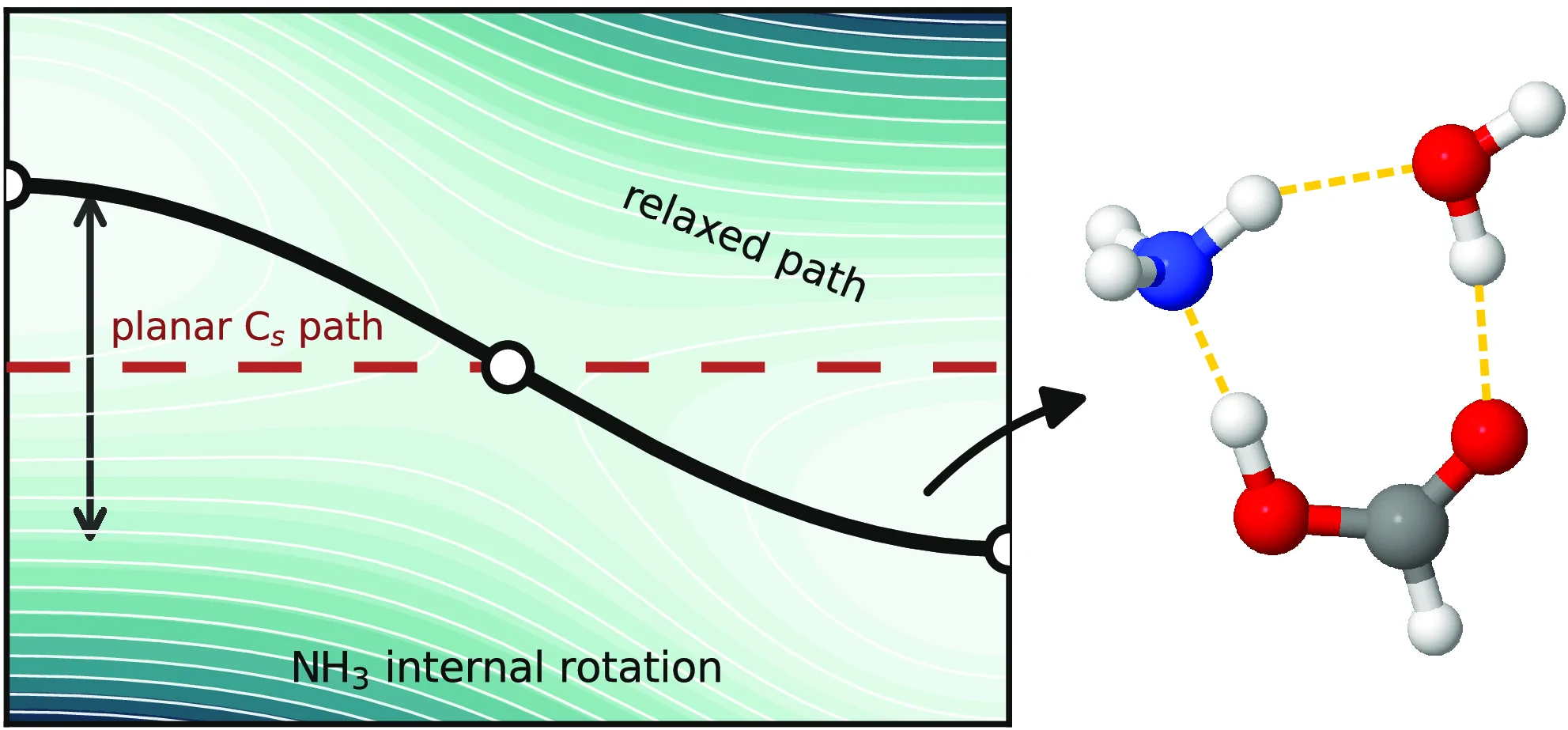

## Introduction

Atmospheric new particle formation is controlled by the first contacts between acids, bases, water, and organic vapors, yet the molecular meaning of “proton sharing” in such clusters remains difficult to quantify.^1–12^ At these earliest stages, cluster growth is controlled not only by intermolecular stabilization, but also by electronic redistribution along acid–base hydrogen bonds. A central question is therefore whether one water molecule merely solvates a preexisting acid–base contact, or whether it can already drive the pair toward incipient proton transfer.

High-resolution rotational spectroscopy provides a powerful route to this question because it isolates weakly bound clusters one isomer at a time and resolves both their structures and large-amplitude dynamics. When a quadrupolar nucleus is present, hyperfine structure and nuclear quadrupole coupling constants offer an additional experimental probe of local electronic structure.^13–28^ Nuclear quadrupole coupling constants have already proven useful for tracking aggregation-induced electronic redistribution, from ion-pair formation in hydrated strong acids to gradual ionicity development in microsolvated bases. These studies establish quadrupole tensors as sensitive probes of aggregation-induced electronic-structure change, but they leave unresolved the earliest microsolvation limit in a chemically relevant ternary acid–base–water system: can a single water molecule measurably promote proton-transfer character in a neutral acid–base complex?

A quantitative answer requires theory not only to support spectral assignment, but also to convert spectroscopic observables into chemically meaningful descriptors of proton-transfer character. In the present work, theory therefore plays distinct and complementary roles. A density-functional entry level defines the conformational assignment space and guides the initial carrier identification, whereas calibrated high-level structure and vibration models provide the quantitative reference needed to interpret small but chemically significant distortions of the acid–base contact. Chemically matched binary controls further anchor this interpretation, allowing the structural and electronic consequences of adding one water molecule to be distinguished from the intrinsic properties of the parent acid–base pair.

Here we apply this combined experimental–theoretical strategy to the HCOOH–NH_3_–H_2_O cluster by linking three observables: rotational constants, the large-amplitude tunneling pattern, and the ^14^N quadrupole tensor. The tunneling pattern is not a spectroscopic complication, instead the resolved NH_3_ internal rotation supplies the orientation needed to transform the experimentally fitted ^14^N quadrupole tensor into the local ammonia frame. This makes χ_*zz*_ an experimental reporter of proton-transfer character rather than a semiexperimental quantity dependent on calculated off-diagonal tensor elements. The resulting ^14^N tensor shows that the water-induced structural reshaping is electronic as well as geometric: ammonia remains the primary proton acceptor from formic acid, while water acts as a cooperative partner that reorganizes the acid–base contact. The ternary complex reaches about 26% quadrupole-derived ionicity without forming a separated ion pair, demonstrating that one water molecule can shift a neutral acid–base complex toward proton transfer by reshaping the contact itself rather than simply stabilizing a fixed binary structure.

## Computational Details and Structural Framework

This section defines the L0–L4 computational levels and their respective roles in assignment, structural calibration, and proton-sharing analysis. Equilibrium geometries are obtained with several Pisa composite scheme (PCS) variants,^29,30^ which employ CCSD­(T*)-F12*/cc-pVnZ-F12 ^31,32^ frozen core computations plus an additive MP2 (PCS2 and PCS3) or CCSD­(T)^33^ (PCS4) core–valence contribution evaluated with the cc-pWCTZ^34^ basis set. Vibrational corrections are computed at the DPCS3 level, denoted L1 below,^35^ using revDSD-PBEP86-D4 ^36^ with the 3F12^–^ basis set.^37^ This basis is derived from cc-pVTZ-F12 ^38^ by replacing the pair of functions on non-hydrogen atoms with a single f function from cc-pVTZ.^38^ All geometry optimizations use a recently developed external interface^39,40^ that links the Gaussian^41^ geometry driver to Molpro^42^ energy evaluations.

The levels used throughout the text are defined in Table 1. L0 is used only for conformational exploration and initial assignment, whereas L2–L4 provide the structural sequence. In the notation L*i*//L*j*, the equilibrium geometry is optimized at level L*i*, while properties and vibrational corrections are evaluated at level L*j*. Thus, L*i*//L1 denotes an L*i* equilibrium structure combined with L1 corrections and properties for the ground-state comparison.

**Table 1:** Operational Levels Used in the Structural Interpretation

Level	Practical implementation	Role in this work
L0	B3LYP-based entry level	conformational search and assignment support only
L1	DPCS3: revDSD-PBEP86-D4/3F12^–^	anharmonic corrections used in *B*_0_ predictions
L2	PCS2: CCSD(T)-F12/2F12^+^ + MP2/wc3	compact coupled-cluster structural level
L3	PCS3: CCSD(T)-F12/3F12 + MP2/wc3	enlarged-valence structural level
L4	PCS4: CCSD(T)-F12/4F12 + CCSD(T)/wc3	extended-basis benchmark

The conformational search was organized as a topology enumeration rather than an unconstrained stochastic search. The three monomers were combined in all chemically distinct acid–base–water arrangements that preserve reasonable hydrogen-bond valences: ammonia accepting from the acid, ammonia donating to water or to the carbonyl oxygen, water bound to the carbonyl group, water inserted between acid and base, and open or cyclic variants of these patterns. Trial structures were optimized at L0, and duplicate minima were removed by comparing connectivity, rotational constants, and hydrogen-bond distances. The remaining energetically low-lying candidates were then reoptimized through the PCS hierarchy. L0 therefore defines the chemically complete assignment space, whereas the PCS levels define the quantitative structural comparison. The large energy gap of the observed form is a result of this sampled topology space, not of starting from a single guessed structure.

The chemically relevant controls are summarized in Figure 1. They clarify the use of each level: L0 supplies the assignment space, whereas the calibrated PCS sequence tests whether the structural comparison is precise enough for a proton-sharing interpretation. Ordinary neutral dimers and the formic-acid–ammonia acid–base pair do not stress the computational hierarchy in the same way. For simple dimers, intermolecular heavy-atom distances are already stable to within about 1 mÅ from L2 upward, corresponding to about 0.1% in the rotational constants.^35^ Once formic acid donates to ammonia, the acid–base contact becomes more demanding: improving the equilibrium structure from L2 to L4, while keeping the same L1 vibrational correction, lowers the rotational residuals into the 0.1% regime.

**Figure 1:**
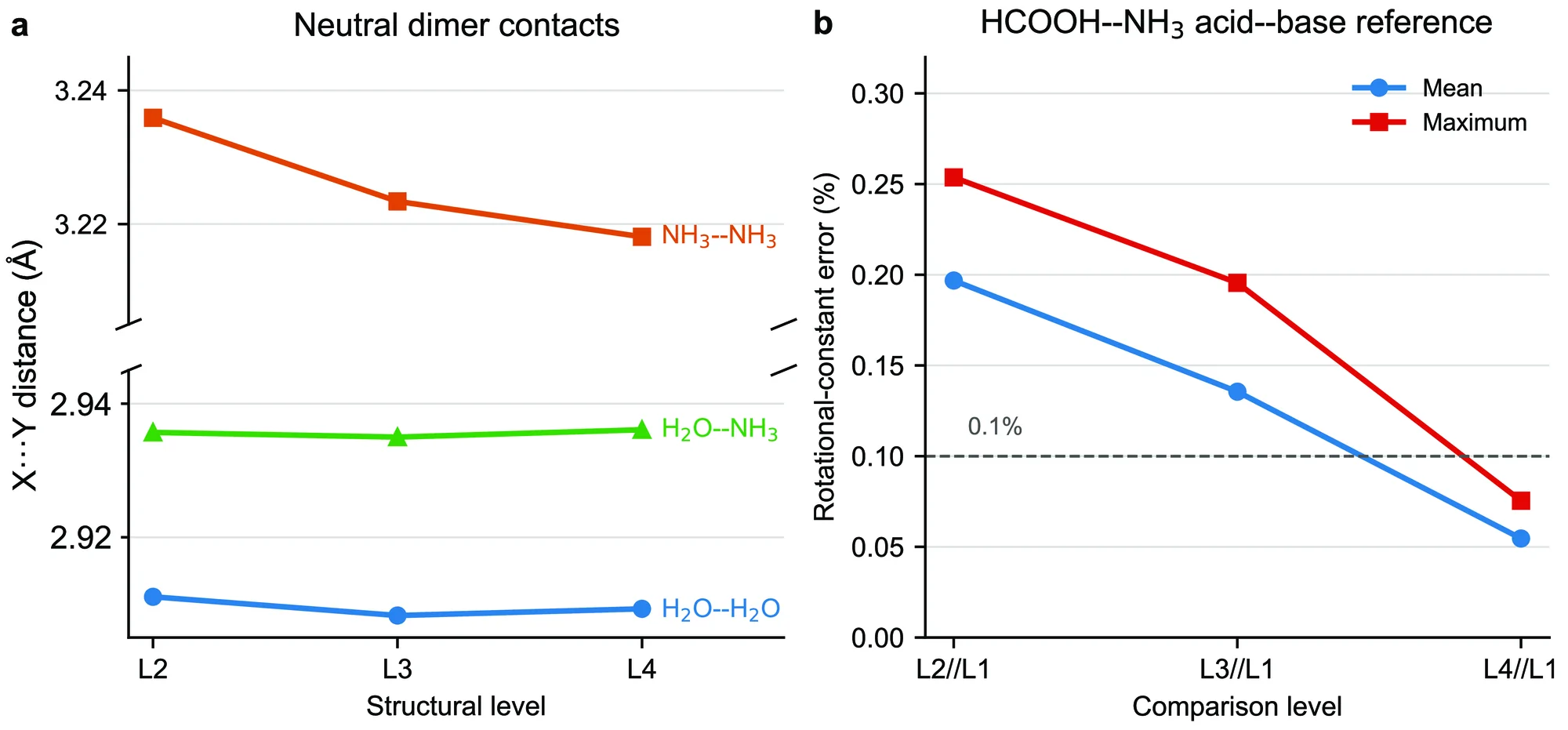
Control benchmarks defining the accuracy required for the ternary interpretation. (a) Intermolecular heavy-atom X···Y distances for H_2_O–H_2_O, NH_3_–NH_3_, and H_2_O–NH_3_ from L2 to L4; the broken vertical axis separates the ammonia dimer from the two shorter contacts. (b) Mean and maximum unsigned relative errors of the HCOOH–NH_3_ rotational constants after applying the same L1 vibrational correction to each equilibrium structure. The dashed line marks the 0.1% spectroscopic window. Detailed numerical values used to generate the figure are given in the Supporting Information.

The topology of the ternary minimum follows the same chemical ordering (Figure 2). It is also consistent with the known binary motifs of formic acid with NH_3_ and with H_2_O, and with the greater basicity of NH_3_ relative to H_2_O. Thus, ammonia remains the electron-pair donor and hydrogen-bond acceptor toward the formic-acid OH group, while water reinforces that acid–base contact rather than replacing it: it accepts from NH_3_ and donates to the carbonyl oxygen, closing a cooperative three-body network.

**Figure 2:**
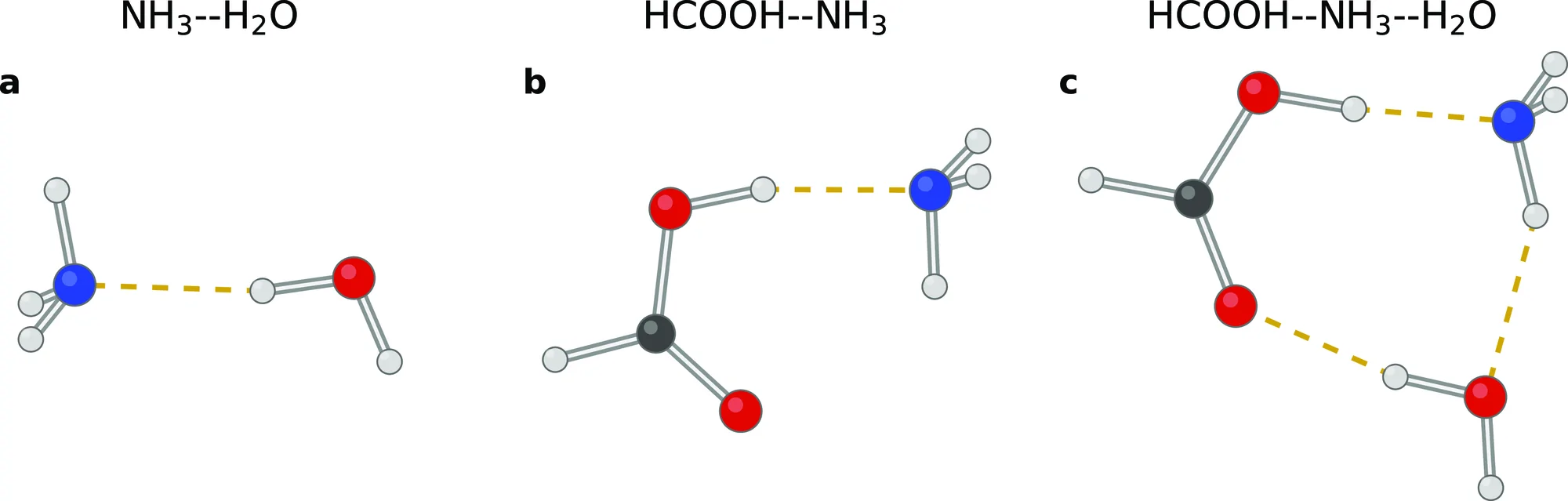
Structural motifs connecting the control systems to the ternary minimum. NH_3_–H_2_O defines the water–base contact, HCOOH–NH_3_ defines the acid–base proton-sharing reference, and the ternary complex combines both while retaining NH_3_ as the primary acceptor from formic acid. Dashed yellow contacts indicate hydrogen bonds; all panels use the same graphical scale.

This structural response gives a chemical rationale for the hyperfine result. Its significance is clearest in the context of the rotational benchmarks. Agreement at about 0.1% in rotational constants corresponds to a root-mean-square (rms) uncertainty of the order of 1 mÅ in atomic positions, providing sufficient resolution for the subtle structural changes associated with proton sharing.^35,43^ The L4//L1 residuals place the structural uncertainty well below the changes induced by one water molecule: the acid O–H bond lengthens by 0.016 Å, the H···N distance shortens by 0.077 Å, and the O···N separation contracts by 0.049 Å (Table 4). These changes are one to two orders of magnitude larger than the residual uncertainty of the calibrated model. They are small on the scale of a full proton transfer, but every coordinate moves in the same direction: toward greater proton sharing by ammonia. The same trend is summarized in the Supporting Information by Δ*r*= *r*(O–H)-*r*(H···N), which increases by 0.093 Å upon addition of water, roughly two orders of magnitude more than the structural uncertainty implied by the rotational benchmarks. The ^14^N quadrupole tensor tests the electronic consequence of this structural shift directly.

The analysis therefore does not rely on qualitative bonding indicators. The proton-sharing enhancement is established from structural and quadrupole observables derived from experiment and interpreted through benchmark-quality L4 structures and L1 vibrational corrections.

## Experimental Methods

The rotational spectrum from 2 to 12 GHz was measured with the COMPACT (compact-passage acquired coherence technique) spectrometer, which implements chirped-pulse Fourier transform microwave (CP-FTMW) spectroscopy.^44–46^ The experimental procedure is summarized below. Approximately 1 g of commercially available ammonium formate was placed in an internal reservoir and heated to 90^◦^C, initiating thermal decomposition to formic acid and ammonia vapor. Simultaneously, 2 mL of tap water was placed in an external reservoir and heated to 37^◦^C to generate water vapor. Neon was used as the carrier gas at a backing pressure of 3 bar. The resulting mixture of diluted formic acid, ammonia, and water vapor was adiabatically expanded into the vacuum chamber, forming a supersonic jet with a rotational temperature of 1–2 K. About 12 million free induction decay (FID) acquisitions were collected and averaged.

## Results and Discussion

The 2–12 GHz broadband spectrum contains a high density of transitions from several species formed under the expansion conditions. Intense signals are assigned to the water hexamer, heptamer, nonamer, and decamer families, (H_2_O)_6–10_,^47–50^ and to ammonia–water complexes, mainly NH_3_–H_2_O and larger NH_3_(H_2_O)_n_ features reported previously under comparable expansion conditions.^51^ Ammonium formate also undergoes a competing decomposition pathway leading to formamide. The intensity of the formamide monomer and its complexes with water^52–54^ increased progressively over the course of the measurements.

After removal of these contributions, a distinct set of rotational transitions remains. These lines show well-resolved doublet splittings and ^14^N hyperfine structure (Figure 3). This pattern is diagnostic of a *C*_*3*_ large-amplitude motion of an ammonia subunit and assigns the carrier to HCOOH–NH_3_–H_2_O. The observed constants select the isomer shown in Figure 4 from the L0 conformational ensemble reported in the Supporting Information. From this point onward, theory has a different role: the assignment-level model identifies the carrier, and the high-level controls define the structural reference for chemical interpretation.

**Figure 3:**
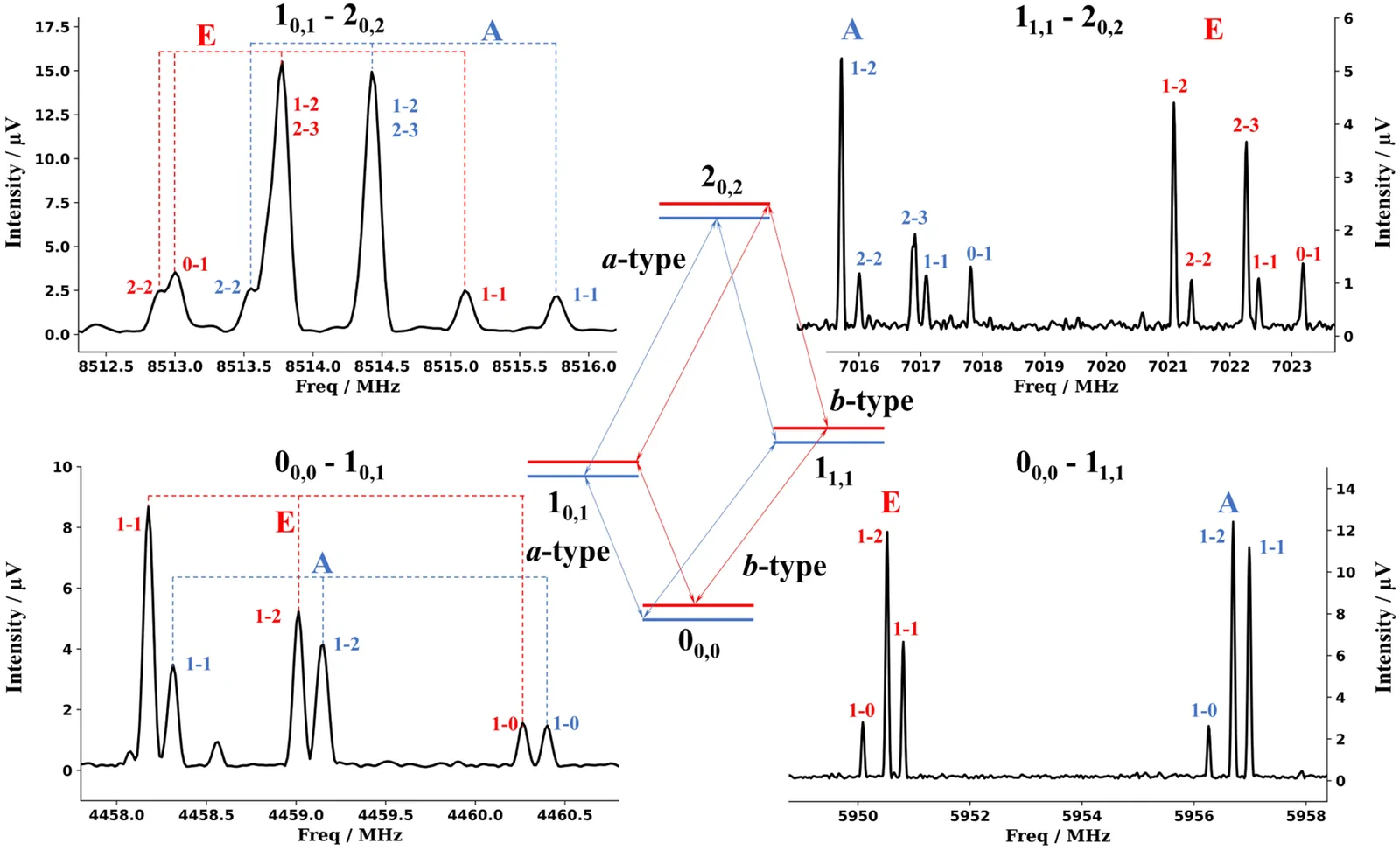
Doublet tunneling splittings with ^14^N hyperfine structure. Rotational states and allowed transitions are indicated in blue and red for the A and E torsional states, respectively. The asymmetric-top levels are labeled as *J_Ka,Kc_*. Hyperfine components are labeled (**F**-**F**’), where **F** = **I** + **J** and **I** = 1 for ^14^N.

**Figure 4:**
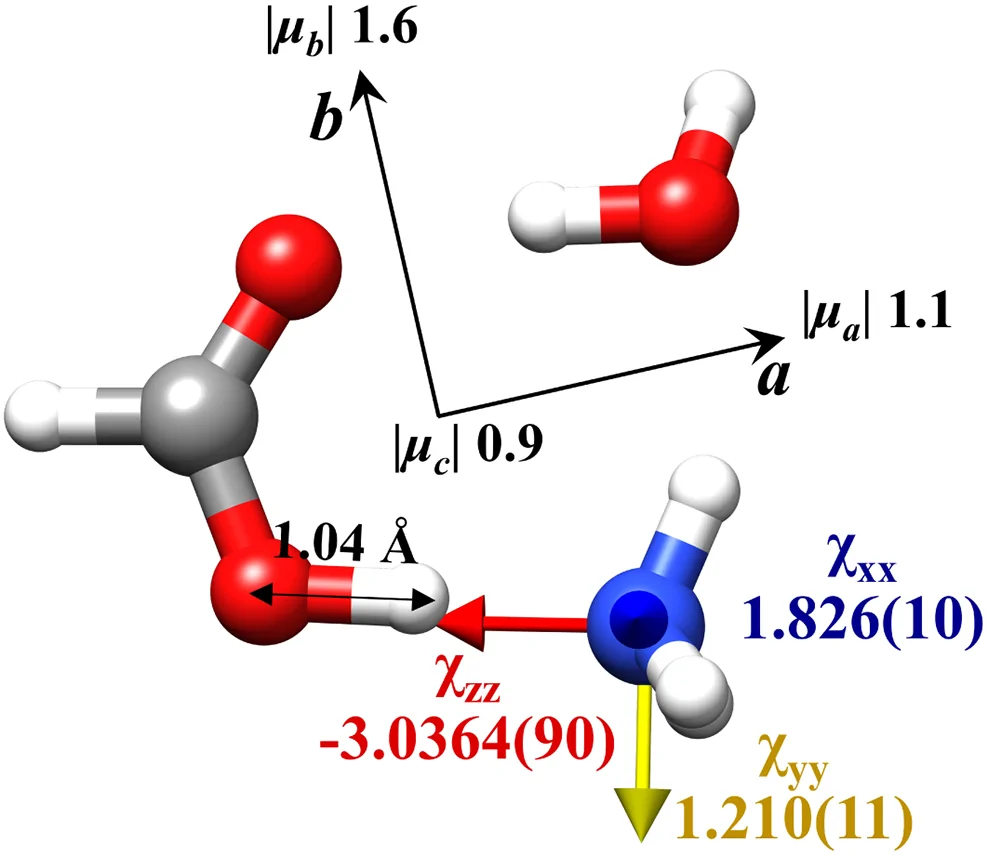
Identified structure of HCOOH–NH_3_–H_2_O in the principal-axis system. Dipole-moment components and the O–H distance calculated at the L0 level are indicated in black in D and Å, respectively. The experimentally determined ^14^N quadrupole coupling constants in the ammonia nuclear-axis system are reported in MHz and colored red, blue, and yellow for χ*_zz_*, χ*_xx_*, and χ*_yy_*, respectively.

The assigned structure has a nearly planar heavy-atom framework and an extended hydrogen-bond network governed by the stronger basicity of NH_3_ relative to H_2_O. From the experimental rotational constants, the planar moment *P*_*cc*_ = (*I*_*a*_ + *I*_*b*_ - *I*_*c*_)/2 is 1.47 amu Å^2^ (Δ = *I*_*c*_-*I*_*a*_-*I*_*b*_ = −2.93 amu Å^2^), consistent with an effectively planar heavy-atom skeleton plus out-of-plane contributions from the hydrogen atoms and large-amplitude averaging. Ammonia is the primary proton acceptor from formic acid, while water links the two partners and reinforces the hydrogen-bond network.

The observed tunneling pattern (Figure 3) reflects two large-amplitude motions. The resolved splitting is assigned to internal rotation of the NH_3_ subunit about its *C*_*3*_ axis. Along this path the strong formic-acid–ammonia hydrogen bond is preserved, whereas the weaker ammonia–water contact is broken and reformed, giving the A/E torsional components characteristic of a 3-fold internal rotor.^55^

The second motion is wagging of the free OH group of water, a common motif in hydrated complexes.^56^ It carries the hydrogen atom through the heavy-atom plane, reversing the sign of the *c*-axis dipole component while leaving the in-plane components unchanged. This motion would generate an additional intrastate splitting for *a*- and *b*-type transitions and an interstate pattern for *c*-type transitions; experimentally, no resolved OH-wagging pattern is detected. This observation is not, by itself, a unique proof of rigid *C*_*s*_ averaging: it is also compatible with population of only the lower wagging tunneling component, with the partner component and associated *c*-type interstate transitions lying outside the observed microwave window. Consistent with this interpretation, the QM treatment places the zero-point level associated with H_2_O libration above the corresponding wagging barrier, so the barrier should not be interpreted as supporting a localized rigid water orientation in the vibrational ground state. This distinction matters for the vibrational treatment. We therefore use the planar *C*_*s*_ geometry as an effective vibrational reference for the ground-state average, rather than as evidence for a static observed structure. In this reference, the inversion coordinate is odd with respect to reflection in the molecular plane, so the pure cubic term along the wagging coordinate is removed by symmetry. The leading correction is therefore quadratic and couples to second derivatives of the moments of inertia, not to their first derivatives. The imaginary frequency at the planar reference is used only as a measure of the local curvature of the large-amplitude coordinate; the physical averaging is governed by the double-well motion.

Only the NH_3_-rotation splittings are resolved experimentally, with magnitudes from 0.6 to 6 MHz (Figure 3). The absence of observable *c*-type transitions, despite the nonzero L0 *μ*_*c*_ component, is therefore treated only as evidence that OH-wagging splittings or interstate transitions are not resolved under the present expansion and frequency-window conditions. It does not by itself distinguish fast averaging from selective population of the lower wagging tunneling state. The A and E torsional states were fitted simultaneously with XIAM.^57^ The resulting rotational, hyperfine, and assignment parameters are summarized in Table 3; the full transition list is available in the data repository.^58^ Quartic centrifugal distortion constants were tested but could not be determined independently because of strong correlations with the tunneling and hyperfine parameters; they were therefore not floated in the final fit.

The fitted internal-rotation barrier is 3.644(62) kJ mol^–1^, with *F* *_0_* = 181.80(32) GHz, corresponding to an effective reduced internal-rotation moment of 2.780(5) amu Å^2^. The barrier is comparable to values reported for pyridine-NH_3_ and glycidol-NH_3_ adducts.^59,60^ The XIAM fit also provides information about the orientation of the interal-rotation axis, which lies in the symmetry plane. The fit includes the two angles ϵ (angle between the b axis and the projection of the internal rotation axis onto the bc plane) and δ (angle between the internal rotation axis and the principal a axis) (Fig. 4): Since the internal rotation axis lies in the ab plane and its projection onto the bc plane is antiparallel to the positive b axis, ϵ is fixed to 180^◦^ and δ is 13.94(15)^◦^. Beyond reproducing the tunneling pattern, the internal-rotation fit connects the spectroscopy to the electronic structure. The fitted NH_3_ rotor-axis orientation defines the transformation from the principal inertial axes to the local ammonia frame, allowing the experimental ^14^N quadrupole tensor to be analyzed along the nitrogen lone-pair direction. This result also clarifies the limited but essential role of the L0 calculations: they guide conformational assignment and predict the observed transition types, but their intermolecular coordinates are not accurate enough for the final proton-sharing interpretation. The quantitative structural discussion is therefore based on the calibrated PCS hierarchy.

The internal-rotation barrier can also be defined on the electronic potential-energy surface. As illustrated schematically in Figure 5, constrained rotation of ammonia is not sufficient, because relaxation of the water wagging coordinate can collapse the structure into an equivalent *C*_*1*_ minimum. The relevant computed quantity is instead the relaxed saddle connecting two ammonia-rotation minima related by a 120^◦^ permutation of the NH_3_ hydrogens. At the L1 level this saddle has one imaginary frequency, dominated by NH_3_ internal rotation, and lies 3.34 kJ mol^–1^ above the corresponding *C*_*1*_ minimum (Table 2). This value is much closer to the XIAM effective barrier than the L0 relaxed-PES estimate, showing that the L1 double-hybrid treatment provides a substantially better description than the L0 assignment-level model for this large-amplitude coordinate. L0 overestimates the barrier by more than 2 kJ mol^–1^. With the experimental *F* value, a one-dimensional 3-fold rotor built from the relaxed L1 barrier gives an A/E ground-state separation of 3.121 GHz, qualitatively consistent with the 2.256 GHz value obtained from the XIAM barrier in the same simplified rotor model. The L0 barrier gives only 0.308 GHz. These rotor splittings should not be compared directly with individual rotational transition doublets, because XIAM determines an effective internal-rotation parameter in a coupled rotational–torsional Hamiltonian. The comparison nevertheless shows that L1 captures both the relaxed torsional barrier and the associated tunneling scale much more reliably than L0.

**Table 2:** Experimental Effective and Calculated Relaxed-PES Quantities for the NH_3_ Internal Rotation^a^

Quantity	XIAM effective fit	L0 relaxed PES	L1 relaxed PES
Barrier/kJ mol^–1^	3.644(62)	5.71	3.34
*F*_0_/GHz	181.80(32)		
*F* ^b^/GHz	186.07	186.07	186.07
Effective reduced moment/amu Å^2^	2.716	–	–
Imaginary frequency/cm^–1^	–	169*i*	127*i*
E_0_(E)-E_0_(A)/GHz	2.256^c^	0.308	3.121

a The A/E separations are obtained from one-dimensional threefold rotors using the experimentally derived *F*; the XIAM entry uses the fitted effective barrier in the same rotor model. Detailed L1 rotor levels are given in the Supporting Information.

b Effective internal-rotation constant derived from the experimental *F*_0_ value in the XIAM fit.

c One-dimensional rotor estimate from the XIAM barrier.

**Figure 5:**
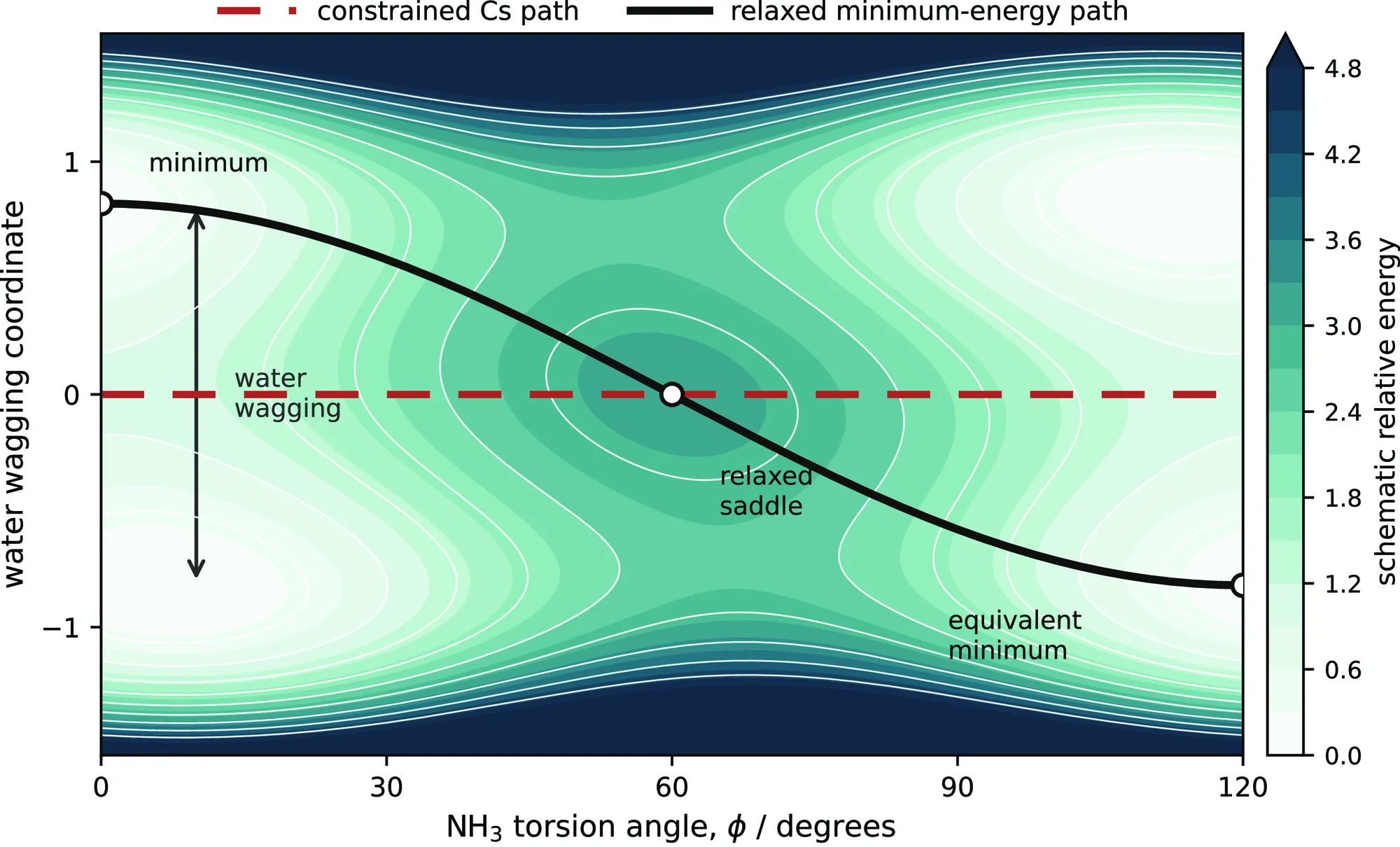
Schematic two-dimensional potential-energy surface connecting water wagging and NH_3_ internal rotation. The dashed path illustrates a constrained planar rotation, whereas the solid path represents the relaxed minimum-energy connection between equivalent *C_1_* minima through the torsional saddle. The surface is qualitative and is used only to define the relevant large-amplitude coordinates.

The calculated constants distinguish assignment-quality agreement from the structural accuracy needed to discuss proton sharing (Table 3). L2 corrects the gross L0 rotational errors, and the L4//L1 ground-state constants bring *B* and *C* below 0.1% and *A* to about 0.2%. The remaining sensitivity is localized in the intermolecular framework, the same coordinate set that controls the O–H/H···N shift in Table 4. The structural calculation therefore has a chemical purpose: it shows that water contracts the acid–base contact before the quadrupole tensor is used to measure the corresponding electronic response.

**Table 3:** Fitted and Calculated Spectroscopic Constants for HCOOH–NH_3_–H_2_O^a^

	Exp	L0 eq.	L2 eq.	L4 eq.	L4//L1
*A*/MHz	4261.4361(39)	4286 (0.6%)	4258(0.1%)	4284(0.5%)	4269.6 (0.2%)
*B*/MHz	2765.4103(43)	2856 (3.3%)	2796(1.1%)	2798(1.2%)	2761.3 (0.1%)
*C*/MHz	1693.5578(28)	1733 (2.3%)	1703(0.6%)	1708(0.9%)	1694.0 (0.03%)
χ_aa_/MHz	–2.7899(72)	–3.35	–	–	–3.19
χ_bb_/MHz	0.964(10)	1.15	–	–	1.09
χ_cc_/MHz	1.826(10)	2.20	–	–	2.10
|μ_a_|, |μ_b_|, |μ_c_|/Debye	|μ_a_| < |μ_b_|, no μ_c_	1.1, 1.6, 0.9	–	–	0.9, 1.3, 0.0

a The fit includes 110 hyperfine components and has an rms deviation of 16.5 kHz. Rotational constants are compared with L0 assignment-level values, selected PCS equilibrium levels, and the L4//L1 ground-state level; calculated ^14^N quadrupole coupling constants and dipole moments are L0 values in the L0 column and L1 properties evaluated on the L4 geometry in the L4//L1 column.

**Table 4:** Selected L4 Equilibrium Proton-Sharing Coordinates for the Binary and Monohydrated Formic-Acid–Ammonia Complexes^a^

System	O–H	H···N	O···N
HCOOH–NH_3_	0.997	1.760	2.743
HCOOH–NH_3_–H_2_O	1.013	1.683	2.694

a Distances are in Å.

The ^14^N nuclear quadrupole coupling tensor provides the electronic counterpart to this structural picture. The χ_*zz*_ component, defined along the nitrogen lone-pair direction, is especially sensitive to depletion of lone-pair electron density and therefore to the ionic contribution to the hydrogen bond.

For an asymmetric top, the experimentally accessible tensor components are expressed in the principal inertial axis system (χ*_aa_*, χ*_bb_*, χ*_cc_*). A chemically meaningful interpretation, however, requires transformation to the nuclear axis system of ammonia. In most cases, this transformation relies on theoretical estimates of the off-diagonal tensor elements, leading to semiexperimental values affected by significant uncertainties.^61^

Here the internal-rotation analysis supplies the missing orientation information. The angle δ = 13.94(15)^◦^ between the internal rotation axis and the principal-axis system defines the transformation from the inertial frame to the ammonia nuclear frame, so χ_*zz*_ can be obtained from experimentally fitted quantities without relying on calculated off-diagonal tensor elements (matrix details are given in the Supporting Information).

This distinction fixes the role of theory in the hyperfine analysis. The L0 and L1 electric-field-gradient calculations in Table 3 support the assignment and tensor orientation, but the ionicity is not taken from those calculations. The quantitative electronic-redistribution metric is the experimentally transformed ^14^N tensor, placed on the Townes–Dailey scale only to compare acid–ammonia complexes on a common experimental basis. Thus, the proton-sharing estimate is derived from a quadrupole tensor obtained experimentally, without theoretical completion of the off-diagonal elements. The Townes–Dailey conversion is used here as an operational scale for comparing ^14^N quadrupole tensors, not as an absolute population analysis or a substitute for the measured hyperfine observable.

The derived χ_*zz*_ value is substantially less negative than that of isolated NH_3_ (χ_0_ = – 4.08983 MHz),^62^ indicating depletion of electron density along the lone-pair direction. On the Townes–Dailey scale,^63–65^

$$i=1-\frac{\chi_{zz}}{\chi_{0}}$$

this corresponds to an ionicity of approximately 26%. The low end of the scale is fixed by H_2_O–NH_3_, for which the measured ^14^N quadrupole interaction is accounted for as a projection of the free-NH_3_ tensor, so the local χ_*zz*_ remains essentially χ_0_ and the corresponding Townes–Dailey ionicity is zero within the operational scale.^66^ The reference HX–NH_3_ series (X = F, Cl, Br, I) provides a useful calibration: within this linear hydrogen-bond family, the quadrupole-derived ionicity tracks the projected proton-sharing coordinate λ_*H*_ = [(**r**_*H*_-**r**_*X*_)·**R̂**_*XN*_]/*R*_*XN*_ (Figure 6). Here **r**_*H*_ and **r**_*X*_ are the position vectors of the bridging H atom and donor atom X, **R̂**_*XN*_ is the unit vector along the X···N donor–acceptor axis, and *R*_*XN*_ is the corresponding donor–acceptor distance. This dimensionless coordinate measures the bridging hydrogen position along that axis; larger λ_*H*_ values indicate displacement of H from the donor X toward the ammonia nitrogen and therefore stronger proton sharing. The bent O–H···N systems define a distinct trajectory: the H_2_O–NH_3_, HCOOH–NH_3_, and HCOOH–NH_3_–H_2_O points are nearly collinear, but they lie on a different line from the HX series because their hydrogen bonds are not linear and the donor atom is oxygen rather than a halogen. This separation shows that the quadrupole-derived metric follows proton sharing within chemically matched families, without transferring the HX–NH_3_ regression to bent formic-acid complexes. Along the bent O–H···N trajectory, addition of one water molecule drives the contact from the weakly ionic binary limit to approximately 26% ionicity, showing that cooperative microsolvation can promote proton sharing beyond the expectation based on intrinsic acid strength alone.

**Figure 6:**
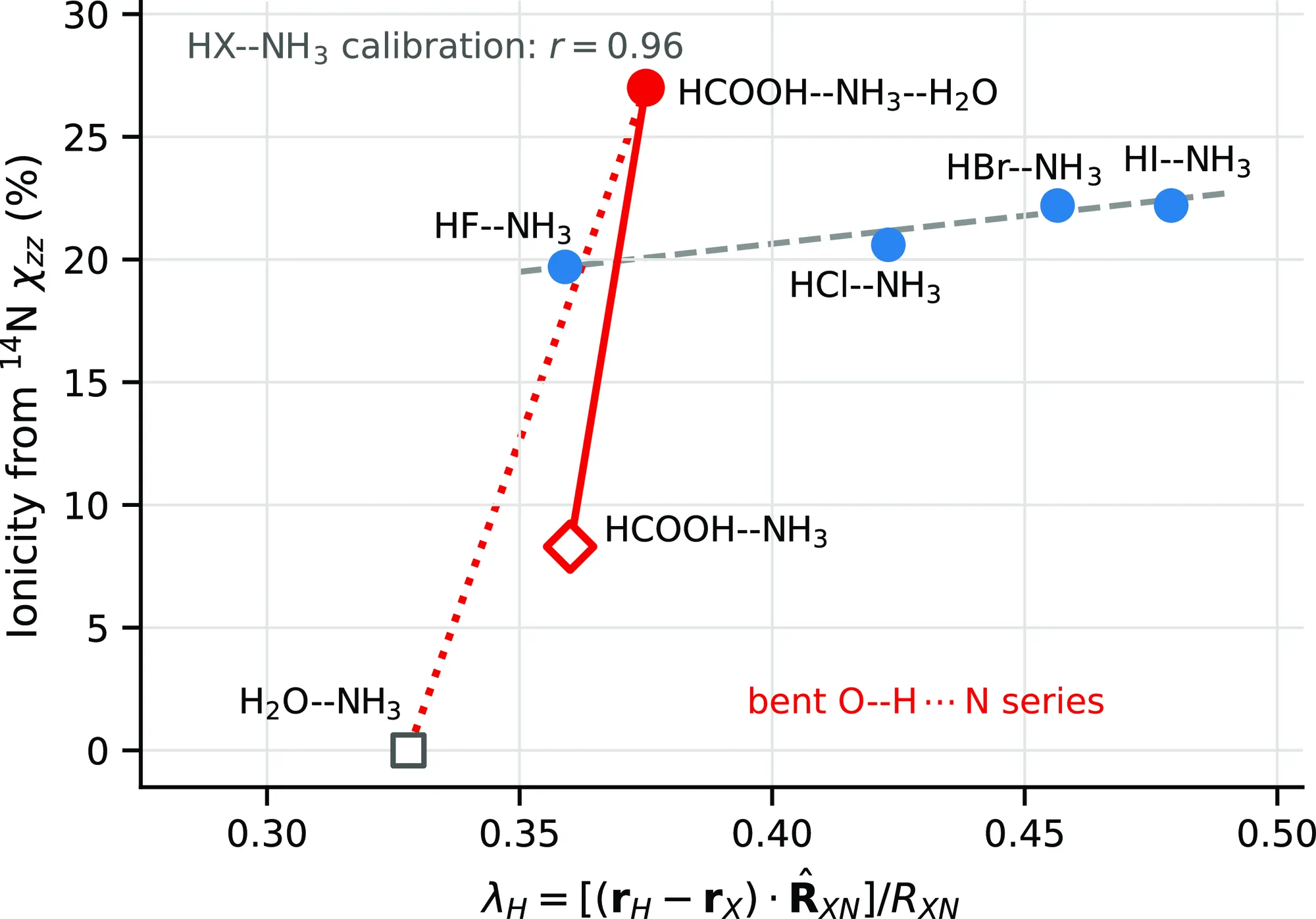
Quadrupole-derived ionicity as a proton-sharing metric. The gray square is the zero-ionicity H_2_O–NH_3_ reference, and the gray dashed line is the least-squares fit to the linear HX–NH_3_ series (X = F, Cl, Br, I; *r* = 0.96). The red dotted line connects the bent O–H···N systems H_2_O–NH_3_, HCOOH–NH_3_, and HCOOH–NH_3_–H_2_O. The open diamond is the semiexperimental HCOOH–NH_3_ value obtained using calculated off-diagonal tensor elements, and the filled red point is the ternary-complex value derived here from the internal-rotation matrix rotation. Ionicities are reported on the Townes–Dailey scale using isolated NH_3_ as the reference, χ_0_ = −4.08983 MHz.^62,66–72^

The enhanced ionicity originates in this three-body arrangement: water accepts from ammonia and donates to the carbonyl oxygen, shortening the acid H···N contact and stabilizing charge-delocalized configurations. Figure 6 shows that the ^14^N quadrupole tensor can act as a quantitative experimental reporter within chemically matched families. The linear HX–NH_3_ complexes define one scale, whereas H_2_O–NH_3_, HCOOH–NH_3_, and the ternary complex define a second, nearly linear O–H···N trajectory. The 26% value is not an isolated Townes–Dailey number; it is the electronic signature of the same cooperative structural shift quantified in Table 4. In this sense, a single water molecule already functions as a proton-sharing promoter rather than a weak perturbation of the binary acid–base pair. The apparent offset from the HX–NH_3_ calibration is not scatter, but chemical specificity of the bent O–H···N family.

An independent natural bond orbital (NBO) descriptor^73^ supports the same conclusion. On the L4 geometries, the NBO analysis gives an LP­(N)→ σ*­(O–H) stabilization of 29.36 kcal mol^–1^ for HCOOH–NH_3_, which rises to 41.25 kcal mol^–1^ in HCOOH–NH_3_–H_2_O (Table S3). This approximately 40% increase in LP­(N)→ σ*­(O–H) stabilization parallels both the structural contraction and the quadrupole-derived increase in proton sharing, providing an independent electronic check on the same water-promoted shift.

## Conclusions

The HCOOH–NH_3_–H_2_O system shows that large-amplitude motion can carry chemical information rather than merely complicate a rotational spectrum. The internal-rotation analysis fixes the orientation of the ^14^N quadrupole tensor, allowing χ_*zz*_ to be obtained experimentally instead of by completing the tensor with calculated off-diagonal elements. This is the key step that turns the ionicity estimate into a measured electronic response of the acid–base contact.

That response shows that one water molecule does more than complete a hydrogen-bond network: it measurably pushes the formic-acid–ammonia contact toward proton sharing. The observed carrier is a single, effectively planar isomer in which NH_3_ remains the primary acceptor from formic acid, and water closes the cooperative network by accepting from ammonia and donating to the carbonyl oxygen. High-level quantum-chemical structures show the same directional response: relative to HCOOH–NH_3_, the acid O–H bond lengthens, the H···N distance shortens, and the O···N separation contracts. The resulting Townes–Dailey ionicity is about 26%, placing the ternary complex on the chemically matched O–H···N trajectory connecting H_2_O–NH_3_, HCOOH–NH_3_, and the monohydrated complex. Thus, water enhances proton sharing in a neutral acid–base pair without crossing into a separated ion-pair limit.

It is important, however, to distinguish the proton sharing observed here from complete proton transfer. Proton sharing denotes displacement and increased delocalization of the proton within the neutral hydrogen bond, whereas proton transfer would require formation of a distinct ionic minimum. In the isolated HCOOH–NH_3_–H_2_O complex, the first water molecule cooperatively reorganizes the hydrogen-bond network and displaces the acidic proton toward ammonia, thereby increasing the proton-transfer character of the neutral acid–base contact. The system nevertheless remains within a single neutral minimum on the potential-energy surface, and no distinct HCOO^–^···NH_4_^+^ minimum is formed. Proton sharing therefore refers here to the displacement and enhanced polarization of the proton within the neutral hydrogen bond. The role of the first water molecule is thus not to induce ion-pair formation, but to reshape the neutral acid–base contact toward proton transfer. Furthermore, rotational spectroscopy probes vibrational-state-averaged rotational constants; under the present conditions, this corresponds to the quantum-mechanical vibrational ground-state average. A finite-temperature description would require state-specific vibrational averages weighted by their thermal populations, which is beyond the scope of the present study. Because hydration lengthens the equilibrium O–H bond and reduces the restoring force without generating a second minimum, it also implies increased proton mobility toward ammonia within the same neutral potential well.

The computational hierarchy makes the conclusions structurally defensible. The entry-level model is sufficient for exploration and assignment, but the final comparison requires L2//L1–L4//L1 treatments because the limiting coordinates are weak intermolecular contacts and their nuclear motion. The same hierarchy is evident for ammonia internal rotation: the L1 relaxed barrier, 3.34 kJ mol^–1^, is close to the XIAM effective value of 3.644(62) kJ mol^–1^, whereas L0 overestimates the barrier and underestimates the tunneling scale. The L4//L1 benchmark is therefore a resolution test rather than a method demonstration: the structural uncertainty must be smaller than the few-hundredths-of-an-angstrom proton-sharing distortion being measured. Together with the binary controls, the result is a rotationally resolved and experimentally quantified example of water-induced proton sharing in the initial microsolvation regime.

## Supplementary Material



## Notes

Conformational survey of the formic acid–ammonia–water complex; rotation of the nuclear quadrupole coupling tensor; proton-sharing coordinate; NBO charge-transfer descriptor; L0 and L1 relaxed internal-rotation barriers; Cartesian coordinates (PDF)
