# Supplementary material for: Single-Water Microsolvation Promotes Proton Sharing in a Neutral Formic Acid–Ammonia Complex
Source: J Am Chem Soc. 2026 Oct 12;148(41):44599–608. doi: 10.1021/jacs.6c13418 (PMC13693395; doi:10.1021/jacs.6c13418)
Supplement: Supplementary file 1 [file ja6c13418_si_001.pdf]

Supporting Information for

Single-Water Microsolvation Promotes Proton Sharing in a Neutral Formic Acid–Ammonia Complex

Fan Xie,* Luigi Crisci, Wenhao Sun, Federico Lazzari, Vincenzo Barone,* and Melanie Schnell*

Fan Xie: Hefei National Research Center for Physical Sciences at the Microscale, University of Science and Technology of China, Hefei, Anhui 230026, China; and Deutsches Elektronen-Synchrotron DESY, Notkestr. 85, 22607 Hamburg, Germany. Email: xiefan@ustc.edu.cn.

Luigi Crisci: Scuola Superiore Meridionale, Largo San Marcellino 10, 80138 Napoli, Italy.

Wenhao Sun: Deutsches Elektronen-Synchrotron DESY, Notkestr. 85, 22607 Hamburg, Germany.

Federico Lazzari: Scuola Superiore Meridionale, Largo San Marcellino 10, 80138 Napoli, Italy.

Vincenzo Barone: INSTM, via G. Giusti 9, 50121 Firenze, Italy. Email: vincebarone52@gmail.com.

Melanie Schnell: Deutsches Elektronen-Synchrotron DESY, Notkestr. 85, 22607 Hamburg, Germany; and Institut für Physikalische Chemie, Christian-Albrechts-Universität zu Kiel, Max-Eyth-Str. 1, 24118 Kiel, Germany. Email: melanie.schnell@desy.de.

Figure Data: Control Benchmarks

The following values were used to generate the control benchmark figure in the Article. The dimer entries are heavy-atom X$\cdots$Y distances. For HCOOH$--$NH$_3$, the errors compare experiment with ground-state rotational constants obtained by adding the same L1 vibrational correction to each equilibrium structure.

System	L2	L3	L4
H_2O--H_2O, O$\cdots$O (Å)	2.9111	2.9083	2.9093
NH_3--NH_3, N$\cdots$N (Å)	3.2359	3.2234	3.2181
H_2O--NH_3, O$\cdots$N (Å)	2.9357	2.9350	2.9361

HCOOH$--$NH$_3$ error (%)	L2//L1	L3//L1	L4//L1
Mean unsigned	0.197	0.136	0.055
Maximum unsigned	0.254	0.196	0.075

S1. L0 Conformational Survey

The initial conformational search was carried out at the B3LYP-D3(BJ)/def2-TZVP level. This level is denoted L0 in the Article and is used only to define the assignment space

and generate starting structures for the high-level PCSn refinements. Table S1 reports the relative zero-point-corrected energies, raw electronic dissociation energies, rotational constants, dipole-moment components, and ^{14}N nuclear quadrupole coupling constants for the four lowest L0 isomers of HCOOH$--$NH$_3$$--H_2$O. The observed carrier is the global minimum of this L0 ensemble.

Table S1: L0 conformational survey for HCOOH$--$NH$_3$$--H_2$O. Relative zero-point-corrected energies (ΔD_0) and raw dissociation energies (ΔD_e) are in kJ mol^{-1}, rotational constants are in MHz, dipole-moment components are in Debye, and quadrupole coupling constants are in MHz. All structures were optimized at the B3LYP-D3(BJ)/def2-TZVP level. The observed isomer is shown in bold.

Isomer	ΔD_0[a]	ΔD_e	A	B	C	$\|\mu_a\|$	$\|\mu_b\|$	$\|\mu_c\|$	χ_{aa}	χ_{bb}	χ_{cc}	χ_{ab}	χ_{ac}	χ_{bc}
**1**	**0.0**	**0.0**	**4286**	**2856**	**1733**	**1.1**	**1.6**	**0.9**	**-3.4**	**1.2**	**2.2**	**0.9**	**0.1**	**0.0**
2	20.4	22.7	9027	1492	1289	4.2	2.5	0.0	1.0	-3.1	2.0	-1.9	0.0	0.0
3	20.5	23.6	4869	2248	1772	2.4	0.1	0.7	0.2	-0.8	0.6	1.9	-1.6	2.0
4	20.5	22.1	8663	1489	1284	4.5	1.7	1.3	1.2	-3.2	2.0	-1.7	0.0	0.0

[a] Relative zero-point-corrected energies are referenced to the global-minimum L0 structure.

S2. Rotation of the ^{14}N Quadrupole Coupling Tensor

The fitted quadrupole coupling tensor is first obtained in the principal inertial axis system (a, b, c). For chemical interpretation, the tensor is transformed into the ammonia nuclear frame. With the axis convention used in the Article, the transformed tensor is written in the (z, y, x) nuclear-axis order, where z is the nitrogen lone-pair axis and x is perpendicular to the local ammonia plane. The transformation is

$$\boldsymbol{\chi}^{(zyx)} = \mathbf{P}\,\boldsymbol{\chi}^{(abc)}\mathbf{P}^{\mathrm{T}}, \tag{1}$$

$$\boldsymbol{\chi}^{(abc)} = \begin{pmatrix} \chi_{aa} & \chi_{ab} & \chi_{ac} \\ \chi_{ab} & \chi_{bb} & \chi_{bc} \\ \chi_{ac} & \chi_{bc} & \chi_{cc} \end{pmatrix}, \qquad \boldsymbol{\chi}^{(zyx)} = \begin{pmatrix} \chi_{zz} & \chi_{zy} & \chi_{zx} \\ \chi_{zy} & \chi_{yy} & \chi_{yx} \\ \chi_{zx} & \chi_{yx} & \chi_{xx} \end{pmatrix}. \tag{2}$$

The rotation matrix is built from rotations about the c, b, and a axes:

$$\mathbf{P} = \mathbf{R}_c(\alpha)\mathbf{R}_b(\beta)\mathbf{R}_a(\gamma), \tag{3}$$

$$\mathbf{R}_c(\alpha) = \begin{pmatrix} \cos\alpha & -\sin\alpha & 0 \\ \sin\alpha & \cos\alpha & 0 \\ 0 & 0 & 1 \end{pmatrix}, \quad \mathbf{R}_b(\beta) = \begin{pmatrix} \cos\beta & 0 & \sin\beta \\ 0 & 1 & 0 \\ -\sin\beta & 0 & \cos\beta \end{pmatrix}, \tag{4}$$

$$\mathbf{R}_a(\gamma) = \begin{pmatrix} 1 & 0 & 0 \\ 0 & \cos\gamma & -\sin\gamma \\ 0 & \sin\gamma & \cos\gamma \end{pmatrix}. \tag{5}$$

For HCOOH−−NH$_3$−−H$_2$O, the internal-rotation analysis gives $\alpha = \delta = 13.94(15)°$, while $\beta = 0°$ and $\gamma = 0°$. The experimental diagonal components in the principal axis system are

$$\chi_{aa} = -2.7899(72)\ \text{MHz}, \qquad \chi_{bb} = 0.964(10)\ \text{MHz}, \qquad \chi_{cc} = 1.826(10)\ \text{MHz}. \tag{6}$$

The off-diagonal components in the principal axis system are not fitted directly. They are obtained by imposing the symmetry condition that the off-diagonal components vanish in the ammonia nuclear frame:

$$\chi_{zy} = \chi_{zx} = \chi_{yx} = 0. \tag{7}$$

Equivalently,

$$\left[\mathbf{P}\boldsymbol{\chi}^{(abc)}\mathbf{P}^{\mathrm{T}}\right]_{zy} = 0, \qquad \left[\mathbf{P}\boldsymbol{\chi}^{(abc)}\mathbf{P}^{\mathrm{T}}\right]_{zx} = 0, \qquad \left[\mathbf{P}\boldsymbol{\chi}^{(abc)}\mathbf{P}^{\mathrm{T}}\right]_{yx} = 0. \tag{8}$$

Solving these three linear equations gives

$$\chi_{ab} = 0.9928(125)\ \text{MHz}, \qquad \chi_{ac} = 0, \qquad \chi_{bc} = 0. \tag{9}$$

The diagonal components in the ammonia nuclear frame are therefore

$$\chi_{zz} = -3.0364(90)\ \text{MHz}, \qquad \chi_{xx} = 1.826(10)\ \text{MHz}, \qquad \chi_{yy} = 1.210(11)\ \text{MHz}. \tag{10}$$

These values are the experimental tensor components used in the Townes–Dailey analysis discussed in the Article.

S3. Proton-Sharing Coordinate

As a compact geometric diagnostic independent of the hyperfine analysis, we define

$$\Delta r = r(\mathrm{O-H}) - r(\mathrm{H \cdots N}), \tag{11}$$

where O–H is the covalent formic-acid bond and H$\cdots$N is the hydrogen-bond distance to ammonia. A less negative value of Δr means that the shared proton has moved further away from oxygen and closer to nitrogen along the acid–base coordinate. At the L4 level,

addition of one water molecule changes Δr from -0.763 Å in HCOOH−−NH_3 to -0.670 Å in HCOOH−−NH_3−−H_2O. This 0.093 Å shift is the geometrical counterpart of the enhanced ^{14}N ionicity discussed in the Article.

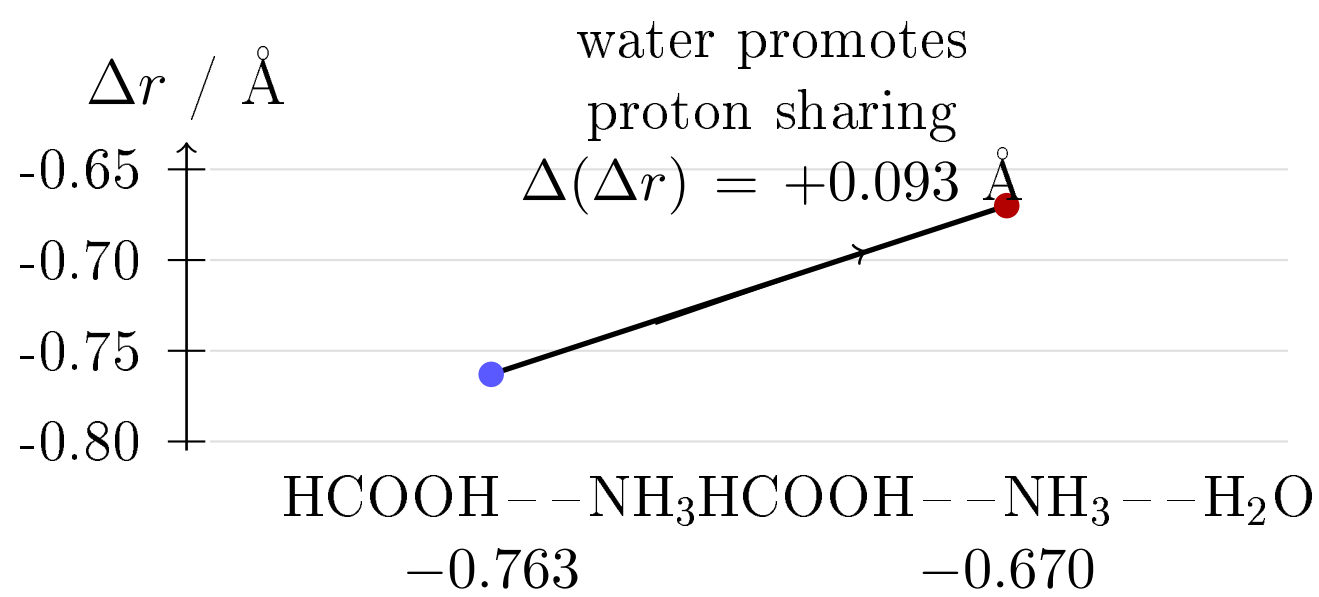

Figure S1: L4 proton-sharing coordinate for the binary acid–base reference and the water-containing ternary complex. The coordinate $\Delta r = r(\mathrm{O-H}) - r(\mathrm{H\cdots N})$ becomes less negative upon addition of water, showing that the acid proton moves toward nitrogen in the initial microsolvation regime.

For comparison with the linear HX−−NH_3 reference series discussed in the Article, we also used a projected proton coordinate,

$$\lambda_H = \frac{(\mathbf{r}_H - \mathbf{r}_X) \cdot \hat{\mathbf{R}}_{XN}}{R_{XN}}, \tag{12}$$

where X is the proton donor atom and $\hat{\mathbf{R}}_{XN}$ is the unit vector along the donor–nitrogen axis. This coordinate reports the fractional position of the acidic hydrogen along the heavy-atom donor–acceptor direction and can therefore be compared across linear HX−−NH_3 complexes. The H_2O−−NH_3, HCOOH−−NH_3, and HCOOH−−NH_3−−H_2O values define the bent O–H$\cdots$N trajectory shown in the Article; they are not included in the HX−−NH_3 calibration fit.

Table S2: Projected proton-sharing coordinate and ^{14}N-based ionicity values used in Figure 6 of the Article. Ionicities are reported on the Townes–Dailey scale using isolated ammonia as the reference.

System	λ_H	χ_{zz} / MHz	Ionicity / %
H_2O−−NH_3	0.328	-4.090	0.0
HF−−NH_3	0.359	-3.283	19.7
HCl−−NH_3	0.423	-3.248	20.6
HBr−−NH_3	0.457	-3.183	22.2
HI−−NH_3	0.479	-3.182	22.2
HCOOH−−NH_3[a]	0.360	-3.75	8.3
HCOOH−−NH_3−−H_2O	0.375	-2.986	27.0

[a] Semi-experimental value from tensor transformation using calculated off-diagonal terms.

S4. NBO Charge-Transfer Descriptor

Natural bond orbital calculations were carried out as B3LYP/def2-TZVP single-point calculations on the L4 geometries used in the Article. The main donor–acceptor term associated with the acid–base proton-sharing coordinate is the donation from the ammonia lone pair into the antibonding orbital of the formic-acid O–H bond, LP(N)→ σ^*(O–H). The NBO result is consistent with the geometrical and hyperfine evidence: addition of one water molecule increases both the second-order stabilization energy and the σ^*(O–H) occupancy (Table S3).

Table S3: B3LYP/def2-TZVP NBO descriptors computed on the L4 geometries. The LP(N)→ σ^*(O–H) interaction is reported as the second-order perturbative stabilization energy $E^{(2)}$. Occupancies are in electrons. Fragment charges are sums of NPA atomic charges.

System	$E^{(2)}_{\mathrm{LP(N)}\to\sigma^*(\mathrm{O-H})}$ kcal mol^{-1}	$n[\sigma^*(\mathrm{O-H})]$ e	$n[\mathrm{LP(N)}]$ e	$q_{\mathrm{NPA}}(\mathrm{NH_3})$ e
$HCOOH--NH_3$	29.36	0.07815	1.92877	+0.063
$HCOOH--NH_3--H_2O$	41.25	0.10381	1.90217	+0.083

The ca. 40% increase in $E^{(2)}$ and the larger population of the O–H antibonding orbital provide an electronic-structure descriptor of the same effect seen experimentally through the ^{14}N quadrupole tensor. The NPA fragment charge on ammonia becomes slightly more positive on going from the binary to the ternary complex, consistent with stronger donation from the nitrogen lone pair into the acid O–H antibonding orbital.

S5. L0 and L1 Relaxed Internal-Rotation Barriers

The relaxed saddle point connecting two equivalent NH_3-rotation minima was optimized at the L1 (DPCS3, revDSD-PBEP86-D4/3F12$^-$) level. The minimum has all real frequencies, whereas the saddle has one imaginary frequency, $127i$ cm^{-1}, dominated by NH_3 internal rotation. The total revDSD-PBEP86-D4 energies are -322.42241030089 hartree for the minimum and -322.42113719866 hartree for the saddle, giving a relaxed electronic barrier of 3.3425 kJ mol^{-1}. For comparison, the corresponding L0 relaxed saddle gives 5.7078 kJ mol^{-1} and an imaginary frequency of $169i$ cm^{-1}. Thus, L1 brings the relaxed electronic barrier much closer to the XIAM effective value of 3.644(62) kJ mol^{-1}.

The one-dimensional threefold rotor levels in Table S4 were obtained from this L1 barrier using the experimental F_0=181.80 GHz. In the same simplified rotor model, the XIAM effective barrier gives an A/E ground-state separation of 2.015 GHz, whereas the L0 relaxed barrier gives 0.268 GHz. The L1 value, 2.799 GHz, therefore follows the experimental effective torsional scale much more closely than L0. These levels refer to an idealized one-dimensional rotor and are used in the Article only to compare the relaxed PES barriers with the XIAM effective internal-rotation parameter.

Table S4: One-dimensional torsional levels from the L1 relaxed threefold barrier. Energies are relative to the A ground torsional level.

Level	GHz	cm^{-1}	kJ mol^{-1}
A_0	0.000	0.000	0.00000
E_0	2.799	0.093	0.00112
E_1	3201.997	106.807	1.27770
A_1	3272.539	109.160	1.30585
A_2	5520.374	184.140	2.20280
E_2	5967.967	199.070	2.38141

S6. Cartesian Coordinates

Coordinates are reported in Å for the L4 structures used in the structural comparison and in Figure 2 of the Article.

NH_3– –H_2O, L4

```
7

N   -1.460175   -0.014174   -0.000000
H   -1.920739   -0.399867   -0.813129
H   -1.920739   -0.399867    0.813129
H   -1.647462    0.979788   -0.000000
O    1.475088    0.057733   -0.000000
H    1.851180   -0.821644   -0.000000
H    0.515226   -0.077727   -0.000000
```

HCOOH– –NH_3, L4

```
9

H    2.259057    1.251445    0.814639
H    2.259057    1.251445   -0.814639
H    1.803014   -0.092345    0.000000
N    1.760374    0.920924    0.000000
C   -1.127166   -0.587218    0.000000
H   -2.186981   -0.860473    0.000000
O   -0.976020    0.726058    0.000000
H    0.000000    0.929044    0.000000
O   -0.235701   -1.401342    0.000000
```

$HCOOH--NH_3--H_2O$, L4

12

H	2.112352	1.514203	0.812973
H	2.112352	1.514203	-0.812973
H	1.909693	0.107986	0.000000
N	1.678009	1.098172	0.000000
H	-0.000000	1.230996	0.000000
C	-1.686995	0.286235	0.000000
H	-2.763470	0.482832	0.000000
O	-0.998831	1.400113	0.000000
O	-1.242414	-0.841749	0.000000
O	1.382404	-1.797991	0.000000
H	0.436040	-1.585574	0.000000
H	1.439666	-2.752242	0.000000
